# Experimental and Numerical Analysis of the Residual Stresses in Seamed Pipe in Dependence on Welding and Metal Forming

**DOI:** 10.3390/ma16062256

**Published:** 2023-03-10

**Authors:** Pavel Solfronk, Jiří Sobotka, Šárka Bukovská, Josef Bradáč

**Affiliations:** Department of Engineering Technology, Faculty of Mechanical Engineering, Technical University of Liberec, Studentská 1402/2, 46117 Liberec, Czech Republic

**Keywords:** virtual factory, numerical simulation, X-ray diffraction, welding, metal forming, residual stresses, Sysweld, PAM-Stamp 2G, weld zone, heat affected zone

## Abstract

Concerning the increasingly widespread utilization of the finite element method (FEM), the concept of the so-called virtual factory is also gaining ground, and not only in the engineering industry. This approach does not use numerical simulations of individual production technologies separately but treats the entire production process as a chain of interrelated technologies. Thus, the output data from one technology is taken as input data for the following technology. The resulting thermal and mechanical effects are then not only dealt with within one technology but always comprehensively within the production process. In the consideration of the loading and subsequent service lives of manufactured components, values of residual stresses are one of the very important characteristics. For these reasons, this paper deals with the effect of residual stresses’ magnitude and distribution during the formation and the final springback of the seamed pipe end section with and without respect to the influence of the preceding welding. The resulting residual stress values from numerical simulations are subsequently compared with the actual values of residual stresses experimentally measured using X-ray diffraction.

## 1. Introduction

The general effort to account for the effects of various technological operations within a complex whole in numerical simulations is currently a major challenge in the pre-production process. In this case, there is a considerable influence of residual stresses after welding (especially in the heat-affected zone—HAZ) on the accuracy of metal forming numerical simulations. That is why two specialized FEM software programs (Sysweld—v.2021 for welding and PAM-Stamp 2G—v2022.0 for metal forming) were linked in this paper.

Nowadays, the utilization of the FEM represents quite a common approach to solving a substantially wide range of different engineering problems. All of the theoretical aspects of the FEM in light of the engineering industry, such as math equations, basic concepts and reviews of the mathematics and mechanics of materials, are given in the work of Kim et al. [1]. Highly appropriate applications of the FEM in structural mechanics and thermal sciences can be found in [2,3]. In terms of the focus of this paper, there are important books that describe general simulations in metal forming and heat transfer [4,5].

Regarding the experimental part of this paper, it was necessary to take into account two major steps: the determination (with the help of X-ray diffraction) or prediction (using the FEM) of the residual stresses induced by the welding of the steel pipe, and numerical simulations of the subsequent metal forming, which has to take into account the previous welding process. There are quite a lot of different studies that deal with these problems. However, the vast majority of them take into account these problems separately, i.e., they describe either the welding process or the metal forming process.

Many authors have already focused on the phenomenon of residual stresses in welding technologies because residual stresses are very important issues in welded parts. A general prediction of the welding-induced residual stresses using the FEM has been investigated in [6,7,8,9,10]. The prediction of the residual stresses through the FEM in a laser beam, an electron beam and arc welding when considering welding technologies can be found in [11,12,13,14,15,16,17]. Some studies have already incorporated the prediction of the residual stresses via the FEM and its validation through experimentation. Li et al. [18] investigated the welding-induced residual stresses in P92/SU304 dissimilar metal butt-welded joints using Sysweld software and simultaneously, by the hole-drilling strain gauge method, measured the distribution of the residual stresses on the top and bottom surfaces of the two butt-welded joints. It was proven that predicted welding residual stresses revealed a relatively good agreement with the measured ones. The application of the hole drilling method to compare measured and predicted welding residual stresses was also used in the work of Puymbroeck [19], where it was taken into account its effect on the fatigue life (also studied in [20,21]). In addition to the fatigue properties, great attention is also paid to the heat treatment processes and their influence on the magnitude of the welding-induced residual stresses [22,23,24]. Some authors studied also the influence of the residual stresses on the distortion of welded material [25,26,27]. Highly interesting studies were carried out by García et al. [28] and Pavan et al. [29]; these authors applied isotropic and kinematic hardening models to estimate their influence on the welding residual stress distribution. Predicted results were compared with the experimentally measured data obtained from X-ray diffraction, and it was found that residual stress prediction accuracy depends on the hardening models as well. In light of the accurate estimation and minimalization of residual stresses in welding, there is also an effort to take advantage not only of FEM but also of machine learning (MA) and genetic algorithms (GA)—see [30]. As mentioned before, in addition to the hole-drilling method, the X-ray diffraction method is very often used to experimentally measure the magnitude of welding-induced residual stresses (e.g., [31,32,33,34,35,36,37]). In addition, there is also an effort to improve the efficiency of X-ray welding image defect recognition, so the deep learning network has also sometimes been used [38].

The prediction of the residual stresses is also very important for metal forming processes. However, the majority of authors, e.g., [39,40,41,42,43,44,45], studied this phenomenon as only welding problems or metal forming problems, thus separately and not as two consecutive steps. Some authors [46,47,48] dealt with the determination of residual stress in formed members and predicted results were compared with the experiments (sectioning, hole drilling method and X-ray diffraction). On the other hand, Lee et al. [49] identified manufacturing variables (and the determination of residual stresses was one of them) that are closely related to the surface defects in the exterior panels of large commercial vehicles. They also investigated the most suitable conditions to minimize such defects. However, in all cited studies, residual stresses were taken into account as a major output or as one of the major effects in light of metal forming.

Nevertheless, there are very few papers, where authors have already incorporated residual stress into their own computation of the metal-forming process. Li et al. [50] studied the effect of the machine-induced residual stresses on the springback of creep age-formed AA2050 plates. They proposed and implemented a simplified residual into a finite element model; a commercial FE software program, PAM-Stamp 2G, was used in this case. They concluded that the required springback prediction of the FE model was significantly improved when the effect of the residual stresses was taken into account. The influence of the welding-induced residual stresses on the subsequent fatigue damage analysis is discussed in [51]. In this case, Wang et al. investigated the fatigue of butt-welded joints using a damage mechanics method. First of all, they determined residual stresses through thermomechanical FEA. Subsequently, the residual stresses were superimposed on the fatigue loading, and such welding-induced plastic damage was taken as initial damage via the elasto-plastic fatigue damage model. Finally, they evaluated, predicted and measured the fatigue properties and relaxation of the residual stresses, which were in good agreement.

## 2. Materials and Methods

The affordability of high-end HW computing systems has enabled the rapid development of technological processes and numerical simulations at all levels of production. The application of specialized software for solving individual engineering tasks is nowadays relatively common, and FEM results show a progressive match with the real technological processes. The mentioned method shows high flexibility in different designs of the given problem in a relatively short period of time and thus significantly reduces the cost. For these reasons, there can be observed constant pressure and efforts to create the concept of a so-called Virtual Factory, where individual technological tasks will not be solved separately, but as a chain of successive technological processes with mutual iterations and the possibility of optimal correction of the production parameters leading to the production of the desired product. This concept assumes the mutual transferability of input data and obtained results in all stages of such a solution, which has turned out to be one of the main problems in the implementation and development of this concept in practice.

**Figure 1:**
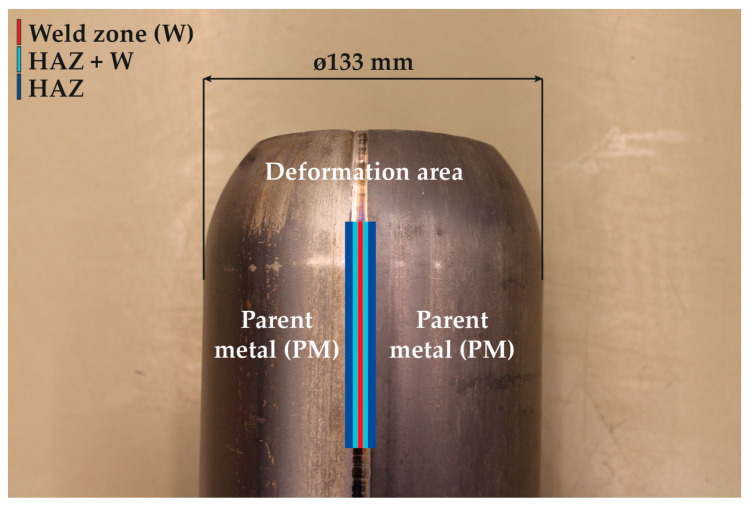
Overview of the analyzed zones (PM, W, HAZ + W and HAZ).

The paper presents the possibility of using a combination of two specialized software programs for the technological processes of welding (Sysweld) and sheet metal forming (PAM-Stamp 2G) developed by ESI Group. For the presentation of the results from experimental measurements and numerical simulations, the forming process of a straight seam welded pipe made from material S355JR with an outer diameter of 133 mm and a wall thickness of 3 mm was chosen. The material used for the production of such pipes is representative of common non-alloy structural steel with low carbon content (max. 0.24 wt%), which is suitable for all types of welding. According to the producer, Huta Pokój Profile Sp. z o.o. (Ruda Śląska, Poland), the joint of the pipe is formed by laser welding without subsequent heat treatment. The shape of the tested pipe with the formed end is shown in Figure 1. In this paper, attention is focused on the analysis of the residual stresses in the formed seam pipe with respect to the influence of both used technologies (welding and metal forming). Values of the residual stresses obtained from numerical simulations are compared with the real values, which are determined through X-ray diffraction. The whole solution process can be summarized as follows:

- Analysis of the tested material mechanical properties to obtain input data for the subsequent numerical simulations;
- Analysis of the residual stresses through X-ray diffraction;
- FEM analysis of the residual stresses for the metal forming in the environment of software PAM-Stamp 2G, marked as **standard FEM**;
- FEM analysis of the residual stresses both for welding and metal forming in the environment of software Sysweld and PAM-Stamp 2G, marked as **modified FEM**.

### 2.1. Static Tensile Test

**Figure 2:**
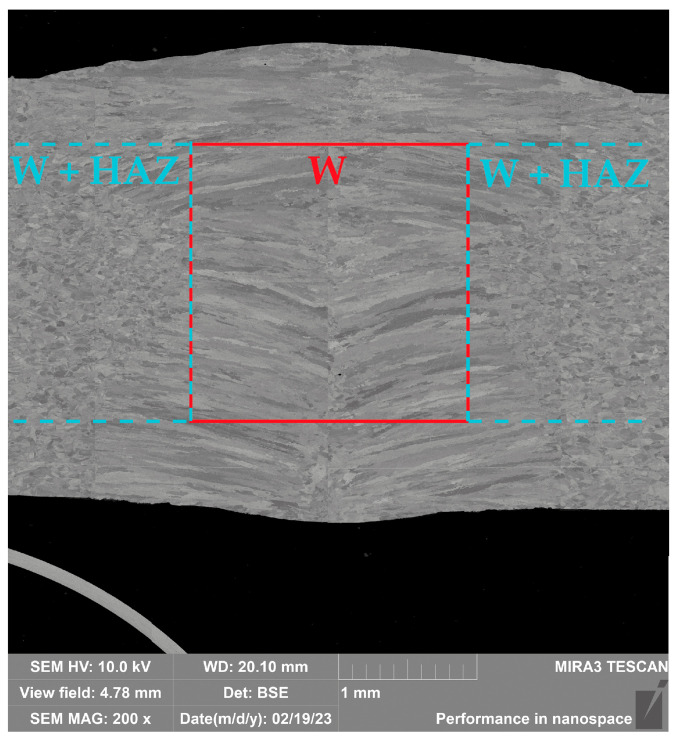
SEM image of the tested laser weld.

**Figure 3:**
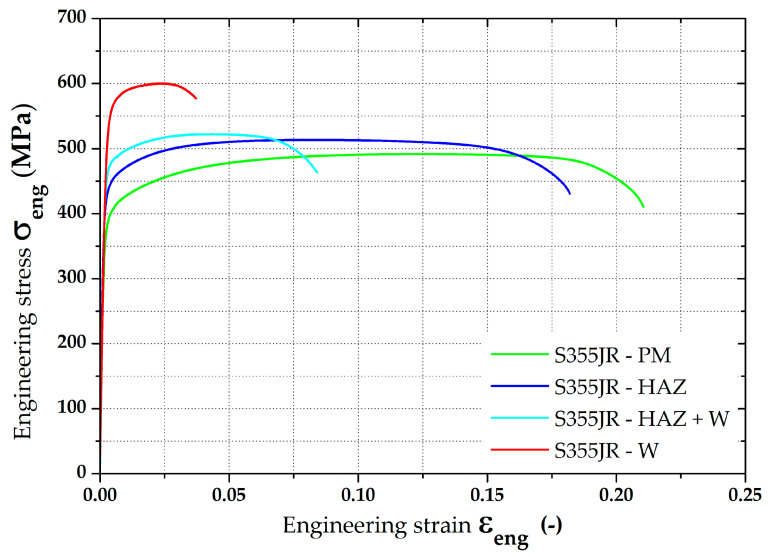
Engineering stress-strain curves (static tensile test) of tested sandwich material.

A static tensile test was used to determine the basic material characteristics of the tested material. With a view of using the experimentally obtained data in future for the definition of material modeling in numerical simulations, these tests have already been performed for samples taken from the parent metal (marked as PM), weld zone (W), zone immediately adjacent to the weld (W + HAZ) and heat-affected zone (HAZ). The locations from where the samples were taken are shown in Figure 1. To eliminate any material anomalies, material characteristic values were determined in each instance from a set of 5 measured samples. When testing the mechanical properties of welds, it is quite difficult to divide the weld zone into precisely defined zones. For this reason, a metallographic scratch pattern of the given weld was made (see Figure 2), which shows how the individual samples were taken for the subsequent tensile testing. For the weld zone (W) and zone immediately adjacent to the weld (W + HAZ), samples were milled and ground to a final square cross-section area of 2 mm × 2 mm (see FIG. X). For the heat-affected zone (HAZ) and parent metal (PM) zones, the samples were only milled to cross-sectional areas of 3 mm × 3 mm (HAZ) and 10 mm × 3 mm (PM), respectively. These samples were then no longer modified in terms of thickness. The gauge length of the test samples was 40 mm. The whole methodology of the test execution and evaluation was in accordance with the standard EN ISO 6892-1. The static tensile test was carried out on a modernized device, TIRA Test 2300, using the software Labtest v.4 to evaluate all basic mechanical properties of tested material (proof yield strength *R_p_*_0.2_, ultimate strength *R_m_*, total ductility *A*_40mm_, uniform ductility *A_g_* and Young’s modulus *E*). The results of the individual material quantities are summarized in Table 1. Engineering stress–strain curves from the static tensile test of tested material S355JR for the individual testing zones are graphically shown in Figure 3. This figure shows averaged curves from 5 measurements.

For a numerical simulation, stress–strain curves (hardening curves) have to be entered as a dependence of true stress (effective stress) vs. true strain (effective strain). Measured dependences of the true stress vs. true strain for the individual rolling directions, 0°, 45° and 90°, were subsequently approximated (fitted) using the Krupkowski law (power–law function) according to Equation (1).

σ = K (ε_pl_ + ε_0_)^n^ (1)

where

σ—true stress (effective stress) (MPa)

K—strength coefficient (MPa)

ε_pl_—true strain (effective strain) (-)

ε_0_—offset true strain (pre-strain) (-)

n—strain hardening exponent (-)

**Figure 4:**
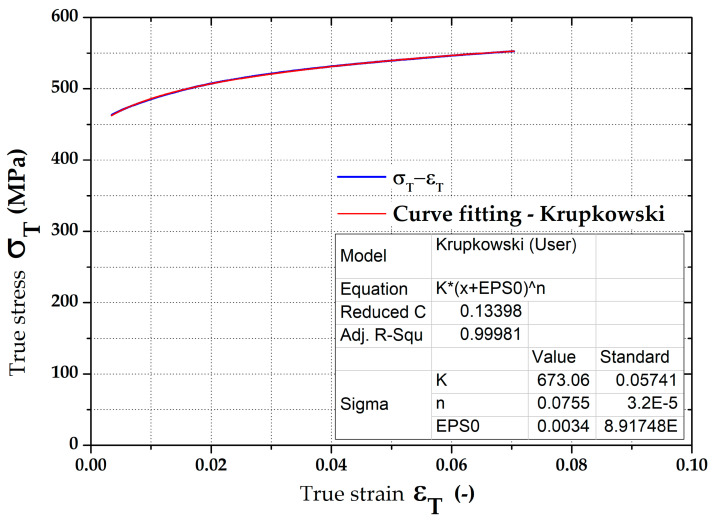
True stress-strain curve (STT) and application of the Krupkowski law.

Approximation of the stress–strain curves (hardening curves) was performed within the interval from 1% up to *A_g_* in the software OriginPro 9.0. The advantage of defining the hardening curve by using the Krupkowski hardening law is the simple possibility of integrating this function, and thus it is easy to express the equivalent plastic work parametrically in dependence on the true strain and also the approximation constants arising from such a function. An example of the approximation result is shown in Figure 4. The determined results of approximation constants are given in Table 2.

### 2.2. Principle of the X-ray Method

X-ray diffraction is based on the scattering of X-rays on crystals of a material. Based on the X-ray scattering, changes are measured in the lattice interplanar spacing (generally d-spacing), which are caused by the applied stress. These changes result in the change in Bragg angle θ (reflection of X-rays). Determined deformations are then recalculated by using equations from the theory of elasticity. Radiation scattering on the adjacent planes leads to an interference maximum in the direction of angle *θ*, if Bragg’s law (2) is satisfied [52,53].

(2) $$n\cdot \lambda ={2d}_{hkl}\cdot sin(\theta )$$

where

n—order of diffraction (-)

λ—wavelength of incident radiation (m)

*d_hkl_*—interplanar spacing of the adjacent lattice planes (m)

*θ*—Bragg angle (-)

The residual stress values in the tested pipes were analyzed via X-ray diffraction (XRD). Device PROTO iXRD COMBO (Proto Manufacturing Inc., Windsor, ON, Canada) was used for the analysis. Tested steel was of ferritic–bainitic structure, and therefore a chromium X-ray tube was used for residual stress analysis (voltage 25 kV, current 4 mA, wavelength Kα = 2.293606 A). Following diffraction elastics, constants of 𝑠_1_ = −1.25 TPa^−1^ and ½𝑠_2_ = 5.75 TPa^−1^ were used to convert deformation to stresses. The diffraction angle value of planes {211} α-Fe material in an undeformed state was 156° 2θ. These values were taken from the database of the software XDR Win2000. The diffraction angles were determined by approximating the Gaussian function using the absolute peak method. The algorithm for calculating the residual stresses was sin^2^ψ. Table 3 shows the basic parameters of the diffraction experiment.

Due to the 1 mm diameter of the collimator, feed of the work desk and oscillation of the X-ray beam, it was only possible to measure residual stresses at points with a minimum distance of 1 mm. Another problem was the curvature of pipe. To achieve a high degree of accuracy in the measured results, the method chosen had the oscillation (displacement) of the work desk occur only in the longitudinal axis of the pipe, thus eliminating its curvature. Four characteristic positions on the pipe, located at 90° intervals, were chosen for residual stress measurements. The starting point, designated as position 0°, is the center of the weld zone.

**Figure 5:**
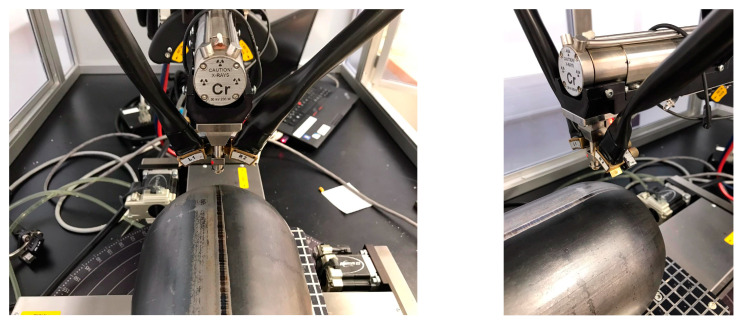
Measurement of the residual stresses via X-ray diffraction.

**Figure 6:**
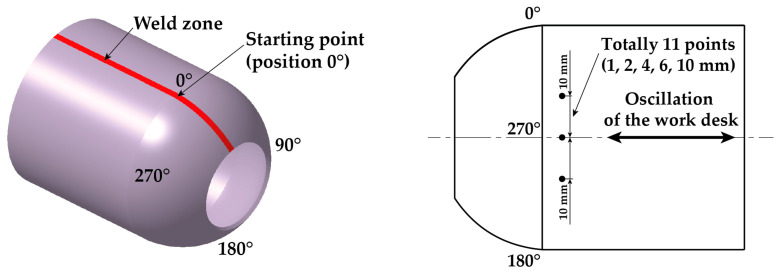
Used measured positions at measurement of residual stress values using XRD method.

The starting point, which was marked as position 0°, is at the center of the weld zone. Around each characteristic point, 5 more points were then measured on both sides at distances of 1, 2, 4, 6 and 10 mm. The method of measuring the residual stresses via X-ray diffraction can be seen in Figure 5 and Figure 6. Four samples were used for the XRD measurements, and the measured residual stress values are shown in Table 4 as the average value from 4 measurements. Since the tested samples were from real industrial production, it was first necessary to remove the surface soil from the pipes without affecting the magnitude of the residual stresses. The XRD measurements were always carried out in a location free of such surface defects. For all 4 measurements, identical experimental conditions (clamping method, position of the scanning point and measurement parameters) were ensured.

## 3. Numerical Simulations

As mentioned in the previous chapter, two different approaches were used to monitor residual stresses. The so-called standard FEM computed residual stresses directly in the PAM-Stamp 2G. On the other hand, the so-called modified FEM took into account results from the previous numerical simulation of welding (software Sysweld).

### 3.1. Numerical Simulation of Welding

The origins of the ESI Group’s Sysweld simulation program date back to the 1980s, when the first software for numerical solutions of welding processes using FEM was developed. This method is based on the approximate solution of partial differential and integral equations. Since the first version of the software was based on a stepwise solution, it always started with the thermal loading and the associated metallurgy and phase transformations, continuing with the mechanical analysis of stresses and strains. Over the years, the software has been continuously improved and extended with new modules. In its current form, it is a comprehensive system covering all interactions between thermal, metallurgical, chemical and mechanical processes.

The program links thermal, electro-kinetic and metallurgical equations. The computations of the mechanical properties are then dependent on the metallurgical history, thermal loading and plasticity. In general, the computations are performed in a given sequence, and the output of one step is the input for the following step. The advantage of this program is that all relevant physical phenomena associated with temperature effects on the material being welded are included in the computations. The material properties are dependent on the temperature, phase and chemical composition.

The program can simulate standard arc welding technologies as well as high-performance welding technologies. It allows the design of 3D volume parts as well as shell components or their combinations. To achieve high-quality results, it is very important to correctly set up the main input parameters, especially the simulation model, heat-source model and input material data. The numerical computation in the software Sysweld is based on two major analyses: thermo-metallurgical and mechanical. Thermo-metallurgical analysis has to be computed first because its basis is the mechanical analysis.

**Figure 7:**
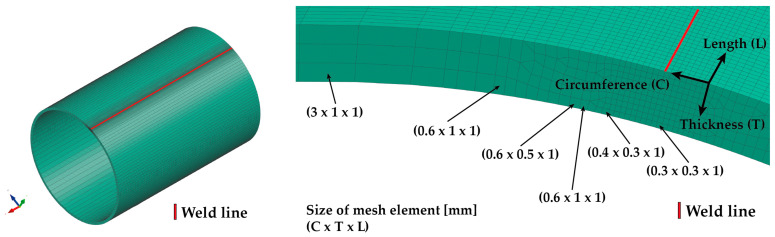
Welding simulation model of pipe and detail of mesh in the weld zone.

In order to perform a simulation computation for welding a pipe of defined dimensions, it is first necessary to create a simulation model that corresponds to the real situation. When creating the finite element mesh for the welding process, a commonly used model meshing strategy was chosen, wherein the weld zone (weld line), a high-density mesh, was created with sufficiently small elements to allow accurate computation. As the distance from the weld increases, the elements become progressively larger. Considering the assumed laser weld width corresponds to approx. 3 mm of sheet thickness, a high-density mesh of 0.3 × 0.3 × 1 mm (circumferential direction × thickness direction × length of pipe) with a total width of 4.8 mm was created in the weld zone. The change in the mesh element’s dimensions with increasing distance from the weld can be seen in Figure 7. The final simulation model contains 126,000 volume elements. The isotropic hardening model was used to compute the residual stresses.

The welding process was performed on the device IPG YLS-2000-S2T (IPG Photonics, Oxford, MA, USA) under the following basic process parameters: power 1800 W, pulse duration 50/5 ms, spot size 1 mm and feed rate 0.01 m·s^−1^. The same conditions were adjusted for the numerical simulation of welding.

**Figure 8:**
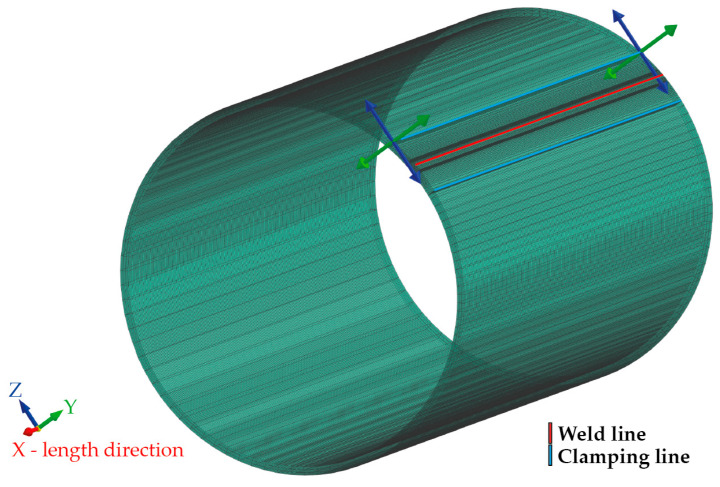
Heat transfer region and definition of simulation model clamping.

Subsequently, the model needed to define the method and area of clamping during welding, which should correspond to the real conditions of the welding process. In fact, the seamed pipe is manufactured by continuous roll bending from a strip of sheet metal. During production, the pipe is fixed during welding using rotary bending tools that allow the sheet to move only in the direction of the pipe length. Regarding this, the boundary conditions during welding were chosen such that a clamping line (in Figure 8 shown in blue) was placed at a distance of 11.3 mm from the weld line (in Figure 8 shown in red). Along the clamping line, displacement in the y and z axes was fixed. Displacement in the x-axis and rotation about all three axes at the location of the clamping line were enabled. The elements in the area between the two blue lines (clamping lines) have no limiting boundary conditions.

After setting up the simulation model, material data were assigned from the extensive library of the simulation program Sysweld, which contains most of the materials used. All material parameters are given here in terms of temperature dependence. For materials undergoing phase transformations, CCT (continuous cooling transformation) and TTT (time–temperature transformation) diagrams are available, which is also the case for the material S355JR, which was used in the submitted paper.

Another important step in the preparation of the simulation computation is setting the heat source parameters, especially the level of heat input corresponding to the real welding process. Based on the temperature loading, it is then possible to compute stresses and strains. The setting of the computation time should be sufficiently long so that the results can be obtained after the welded part has cooled down. This is especially true in the case of residual stress analysis after welding.

**Figure 9:**
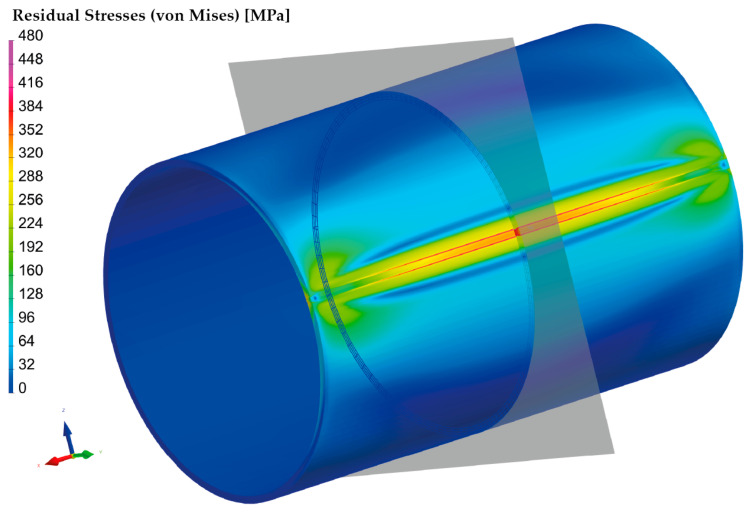
Distribution of the residual stresses (von Mises) after welding and cooling of tested pipe.

According to the procedure mentioned above, the simulation computation of the pipe with a butt weld was set up and subsequently performed. Thermo-metallurgical and then mechanical analysis were computed in sequence. The major aim was to determine the resulting stress state after the welding and cooling of the part. Figure 9 shows the output in the form of the residual stresses according to HMH theory after cooling down. Such theory (in numerical simulation software, often referred to as von Mises) was subsequently used for the computation of all residual stresses. The highest stress values are achieved in the weld zone and HAZ, ranging from 300–470 MPa.

For the numerical simulation of the welding process, a 200-mm-long pipe was chosen. From the result presented in Figure 9, it can be seen that the magnitude of residual stresses at the beginning and end of the pipe is different from that in the middle of the pipe. This is due to the different heat dissipation conditions during welding. Since the pipe production process is, in practice, continuous, the residual stresses after welding were analyzed in the center of the welded pipe, where the welding conditions are already stabilized. The plane section, where these stresses were measured, can be seen in Figure 9.

**Figure 10:**
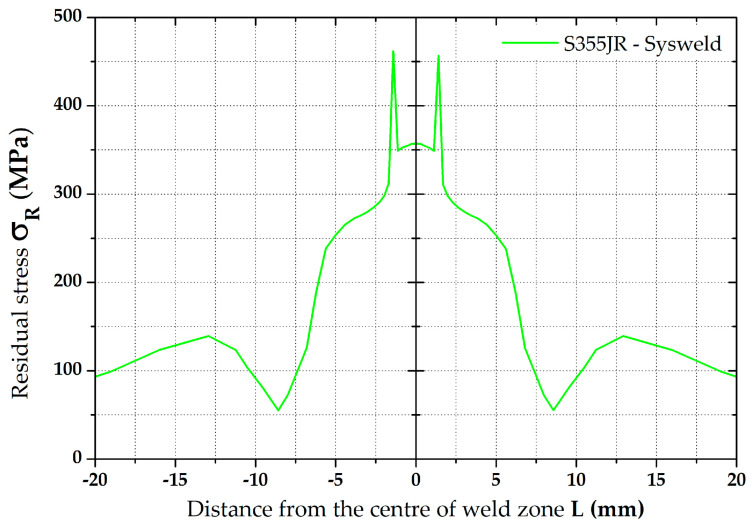
Distribution of residual stresses (von Mises) along section plane after welding and cooling.

A graphical illustration of the residual stress distribution around the weld, obtained by the numerical simulation of welding, is shown in Figure 10.

**Figure 11:**
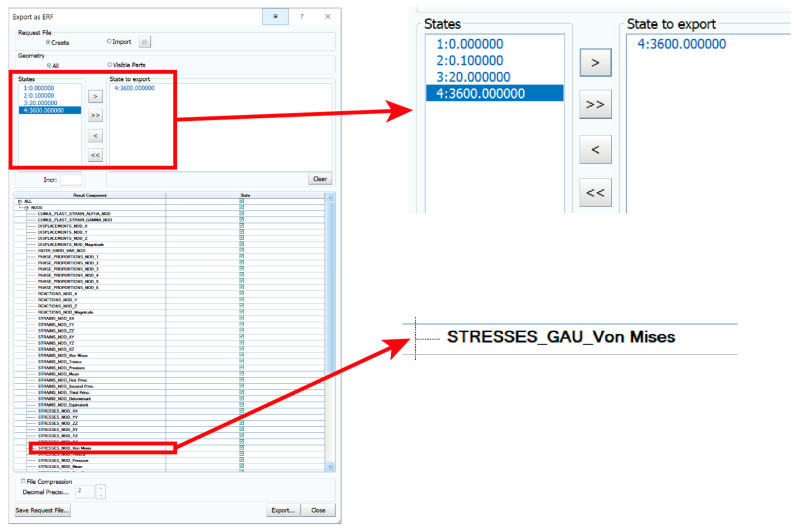
Export of the output data with detail about the required state and residual stress values.

For further use of the residual stresses in the subsequent computations, it was necessary to export these values to a binary file in *.erf format. When exporting, it is possible to select the state to be exported and the required values of the numerical simulation results. In this case, the geometric information of the finite element network and the own values of the residual stresses (von Mises ones) in the state after cooling (State 3600, which is equal to 3600 s) were exported. An example of such an export can be seen in Figure 11.

### 3.2. Numerical Simulation of Metal Forming

The software program PAM-Stamp 2G was used for the metal forming numerical simulation of the pipe end part (see deformation area in Figure 1) with subsequent analysis of residual stresses. For the numerical computation, it was necessary to know the geometry of the tool’s active surfaces, the mechanical properties of the formed material, and the technological conditions of the actual stamping process. The transition area of the tested pipe, which was used for the residual stress analysis, is actually used for the connection of pipes with a smaller diameter in the production of street lighting masts. The dimensions and shape of the forming die were provided by the manufacturer of these masts (Kooperativa Stožáry v.d. (co-opt)) in the form of a CAD file. The real pressing is carried out in such a way that one end of the pipe is fixed (propped) on the machine frame and the other end of the pipe is pressed by a tool (die) placed on a hydraulic piston. The feed rate of the tool during real pressing is 20 mm·s^−1^. The magnitude of the working stroke is about 35 mm. The criterion to terminate the pressing process was obtaining a pipe inner diameter of 91 mm.

**Figure 12:**
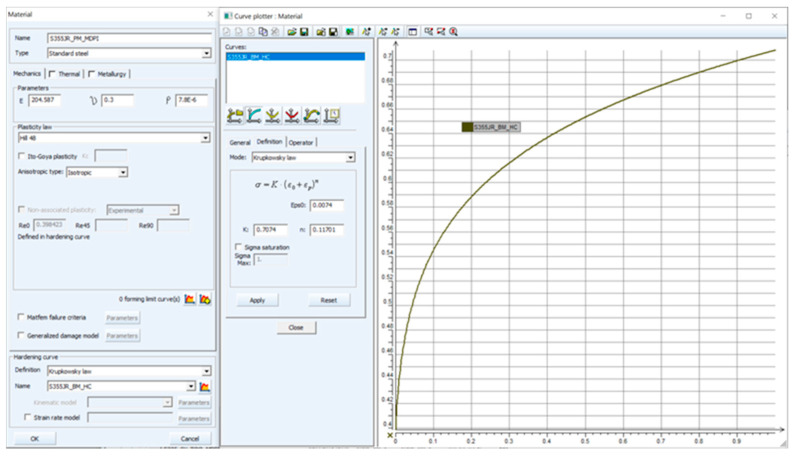
Setting of the stress–strain curve and yield criterion Hill 48.

In the first step of the numerical simulation, the material cards of the formed pipe were defined. The pipe was divided into four regions (PM, HAZ, W + HAZ and W) in which the mechanical properties were investigated as described in the previous chapters. Based on the measured and calculated values (shown in Table 1 and Table 2), material cards were defined in the PAM-Stamp 2G environment. To define a material card, Young’s modulus, Poisson’s ratio, the density (specific mass) and the stress–strain curve had to be entered. The values of Poisson’s ratio and density were taken from the commonly available physics tables. The remaining values needed to fully define the material card were experimentally measured. In addition, the yield criteria had to be selected in the material card. The PAM-Stamp 2G software allows the selection of several major yield criteria. In addition to the commonly used yield criteria, according to Hill (isotropic and anisotropic approach), it is also possible to select so-called advanced yield criteria, e.g., Vegter, Barlat or Yoshida yield criteria. Due to the nature of the formed part (more precisely, the pipe) and the requirement of results that were in accordance with the practice, the isotropic yield criterion according to Hill (rather, Hill 48) was chosen. An example of the material card for the parent metal is shown in Figure 12. Based on the measured data presented in Table 1 and Table 2, the remaining material cards for all tested zones (W, W + HAZ and HAZ) were created analogously.

#### 3.2.1. **Standard FEM**—Analysis of the Residual Stresses Only by PAM-Stamp 2G

The next step of the metal forming process’ numerical simulation was the geometric definition of tools by importing so-called active tool surfaces in the format *.igs with the subsequent automatic discretization of these surfaces. Surface elements with a maximum edge size of 2 mm were chosen for the discretization of the tool surfaces.

**Figure 13:**
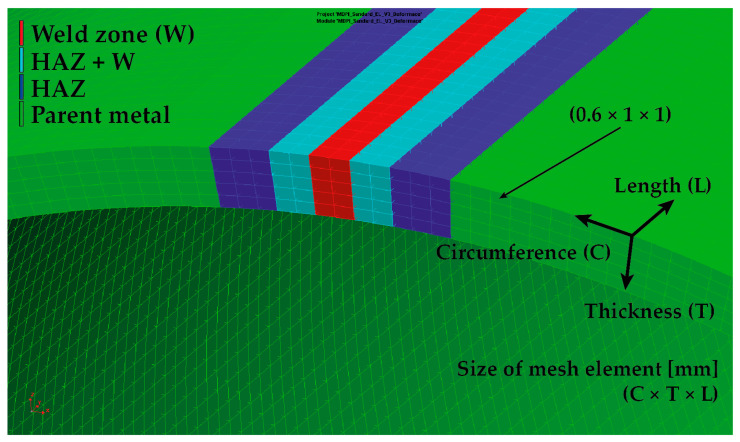
Detail of the applied zones for standard FEM strategy.

A very important step in determining the accuracy and computational time of the metal forming numerical simulation is creating the finite element model of the tested formed pipe. For this purpose, the so-called “Blank editor”, implemented into PAM-Stamp 2G, was used. Regarding the thickness of the pipe (3 mm), volume elements with a constant plane size of 1 × 1 mm were chosen in the Blank editor for computation. In the material thickness direction, five elements with a thickness of 0.6 mm were chosen. Moreover, the pipe was further divided into a weld zone (W) with a width of 2 mm, a zone immediately adjacent to the weld (W + HAZ) with a width of 2 mm and the heat-affected zone (HAZ) with a width of 3 mm. Subsequently, corresponding material cards were assigned to each zone. A detailed view of the finite element mesh used for the computation of the residual stresses can be seen in Figure 13.

**Figure 14:**
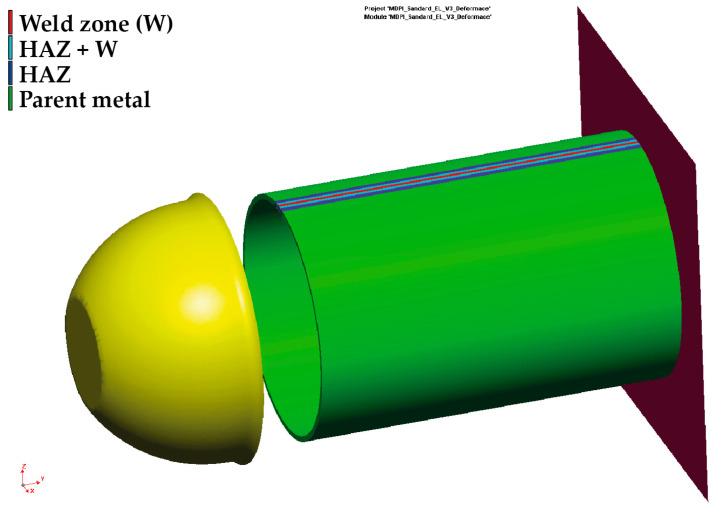
Initial state of all components before the computation.

The initial state of all components before starting the finite element simulation is shown in Figure 14. For the computation, it was necessary to further define the technological boundary conditions as follows: rigid backing plate, movement of the ram with die in the y-axis direction under a constant speed (v = 20 mm·s^−1^) and friction coefficient between contact surfaces (f = 0.12).

**Figure 15:**
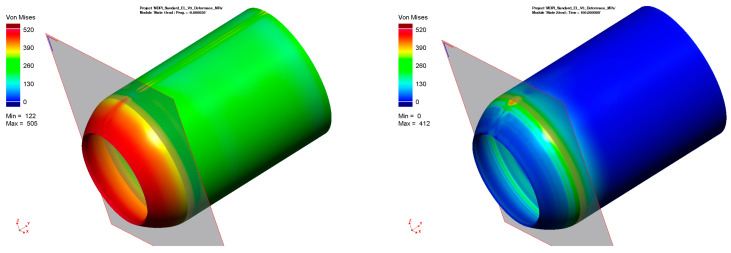
Standard FEM: distribution of residual stresses before (**left**) and after (**right**) springback.

When all of these pre-processing steps have been carried out, the numerical simulation of the stamping process (general processing of data) including the springback of the tested pipe (i.e., both the stamping and springback stages) was performed. The area where the residual stresses after the numerical simulation were measured corresponded with the area where the residual stresses were measured through X-ray diffraction. Strictly speaking, it was the transition area between the deformed and undeformed parts of the pipe. A graphical representation of the residual stresses before and after springback can be seen in Figure 15. In this figure is also shown the position of the section plane, which was used to determine the monitored values of the residual stresses before and after springback. Table 5 shows these values of the residual stresses (FEM after springback), which are compared with the values determined via X-ray diffraction (XRD).

**Figure 16:**
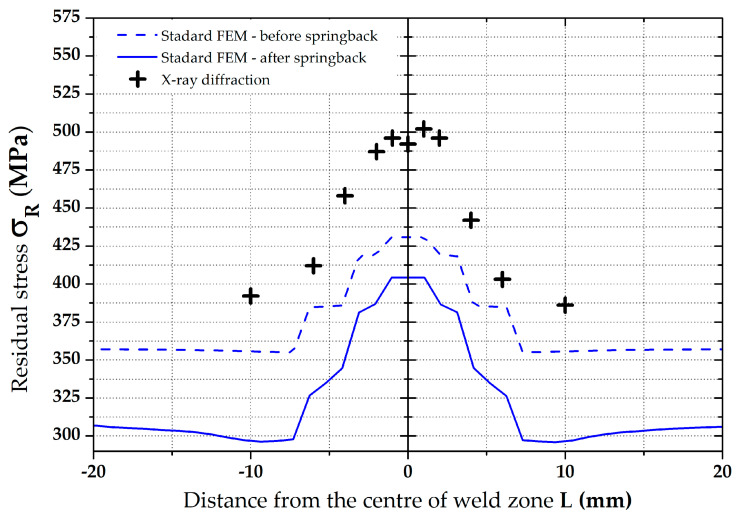
Comparison of residual stresses (von Mises)—XRD vs. standard FEM.

**Figure 23:**
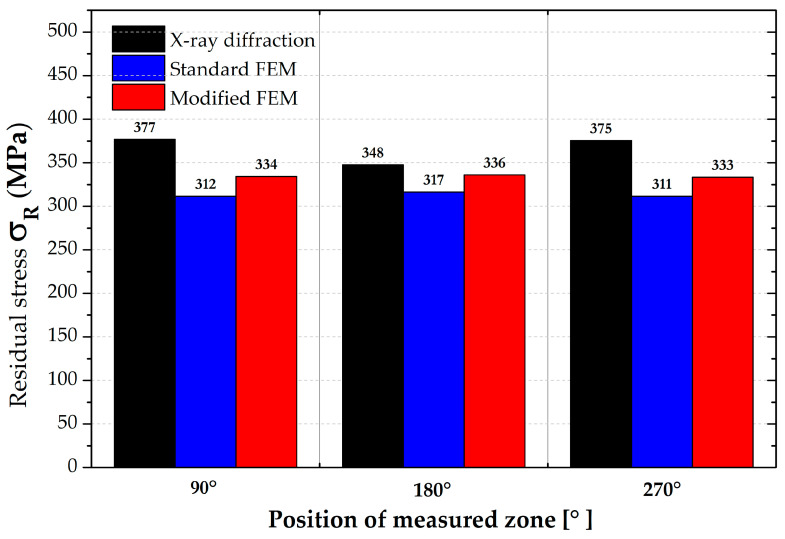
Comparison of residual stresses (von Mises) for measured positions 90°, 180° and 270°.

From the resulting values in Table 5, it can be seen that in the zone around the weld (position 0°), the residual stress values show a significant gradient. In the other monitored zones (positions 90°, 180 and 270°) this phenomenon does not occur, and differences are negligible. Therefore, for the subsequent data evaluation, a graph just for the residual stresses in the position 0° was made—see Figure 16. There can also be found a comparison of FEM data with the measured values. In the other zones, only the average values were subsequently compared—see Figure 23.

#### 3.2.2. **Modified FEM**—Analysis of the Residual Stresses Considering the Previous Welding

**Figure 17:**
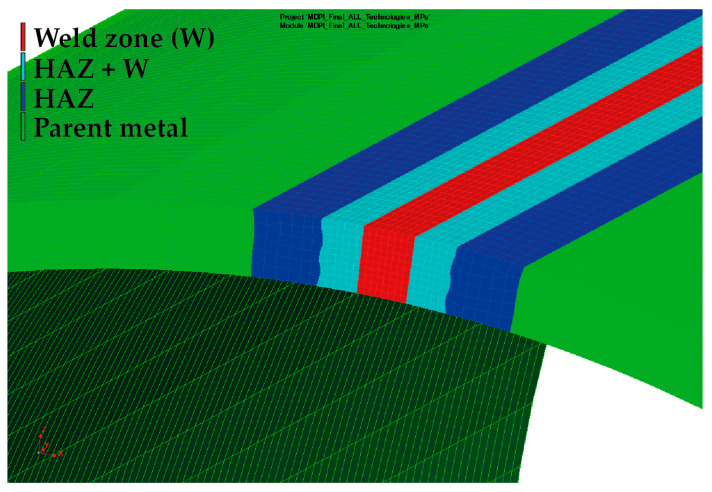
Detail of the applied zones for modified FEM strategy.

From Table 5, it is also obvious that the results from only the numerical simulation of the metal forming process (without considering the influence of the previous welding technology) do not provide sufficiently accurate results for the residual stress distribution in the formed pipe. For this reason, another FEM of the forming process was defined concerning the initial residual stress values arising from the welding process. A necessary condition for the initial transfer of the residual stresses from the welding process to the forming process is the utilization of an identical finite element mesh. With regard to the welding process, where thermal, deformation and stress phenomena occur in small zones around the weld, it is crucial to use the mesh used in the welding part of the numerical simulation. Thus, for the modified metal forming FEM, with respect to the residual stresses after the welding technology (so results from Sysweld), there was also mesh used that was created in Sysweld (see Figure 7). This mesh, including monitored zones, was applied to the software PAM-Stamp 2G (see Figure 17) and finally was used in the modified FEM strategy.

**Figure 18:**
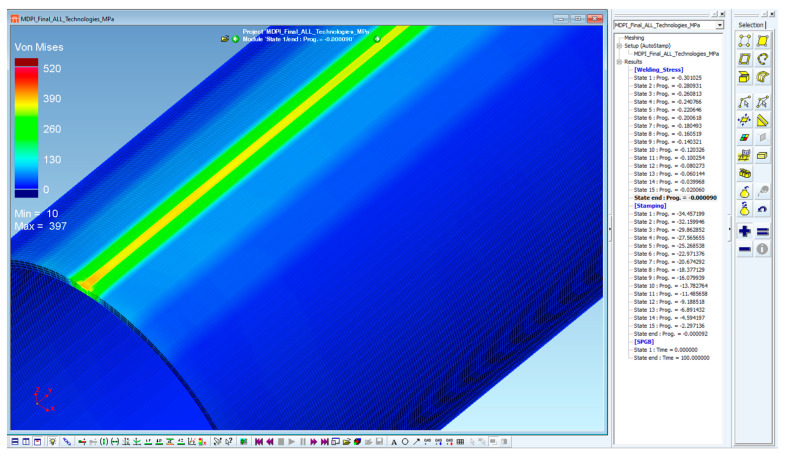
Modified FEM—initial state before the computation of metal forming process.

**Figure 19:**
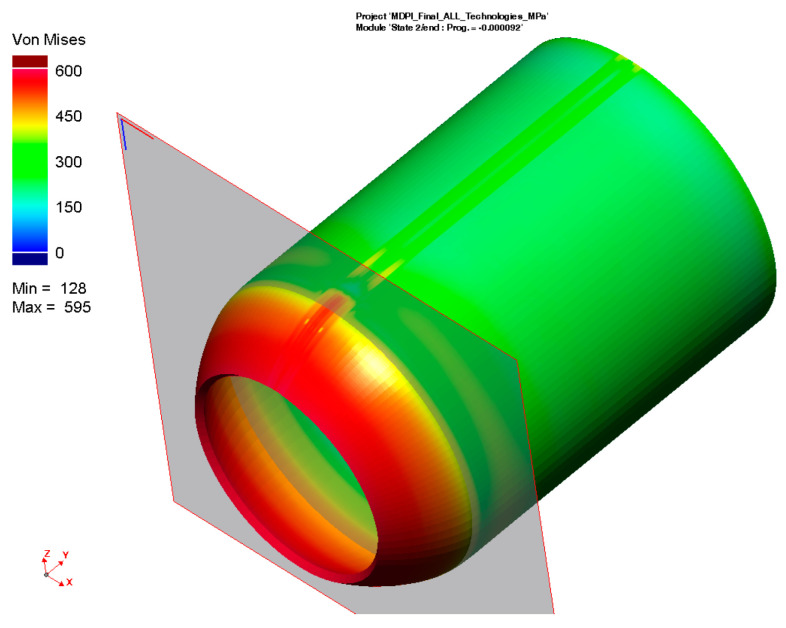
Modified FEM—distribution of residual stresses before springback.

**Figure 20:**
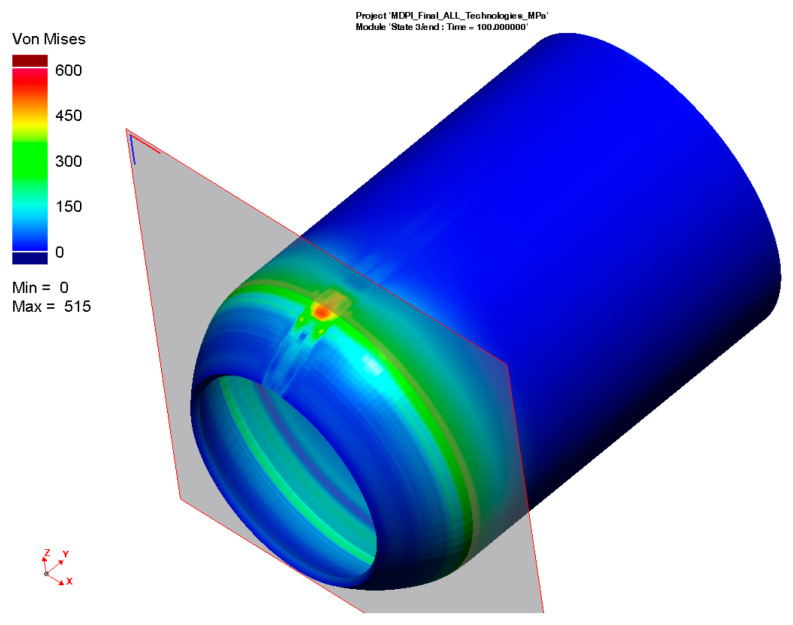
Modified FEM—distribution of residual stresses after springback.

All further steps in the process definition were identical to the previous approach (material card assignment, technological conditions and tool kinematics). Compared to the previous task, an additional auxiliary stage called “welding stress” was created in a new definition of the forming process (modified FEM). Within this stage, the minimum displacement of the die (in this case, 0.3 mm) without contact with the forming material is defined. Thus, there is no influence on the material to be formed. Once the computation is started, the software generates output files for each stage. After the computation of these “welding stress” stages in the software PAM-Stamp 2G, the resulting values of such computations are saved with zero values of stresses, deformations, displacements, etc. This output file in the format *.erf is then integrated with a file obtained from the Sysweld software. So, the stress values from Sysweld are thus implemented into the “welding stress” stages in the environment of the PAM-Stamp 2G. The next step is to start again the numerical simulation of the metal forming (termed as stamping stage), where the input stresses in the volume elements are already taken into account. The distribution of the residual stresses before the actual forming process is shown in Figure 18. Similarly, as in the previous task, residual stress values were monitored in the relevant section plane (see Figure 19 and Figure 20), and their values (again, only after the springback) are given in Table 6.

**Figure 21:**
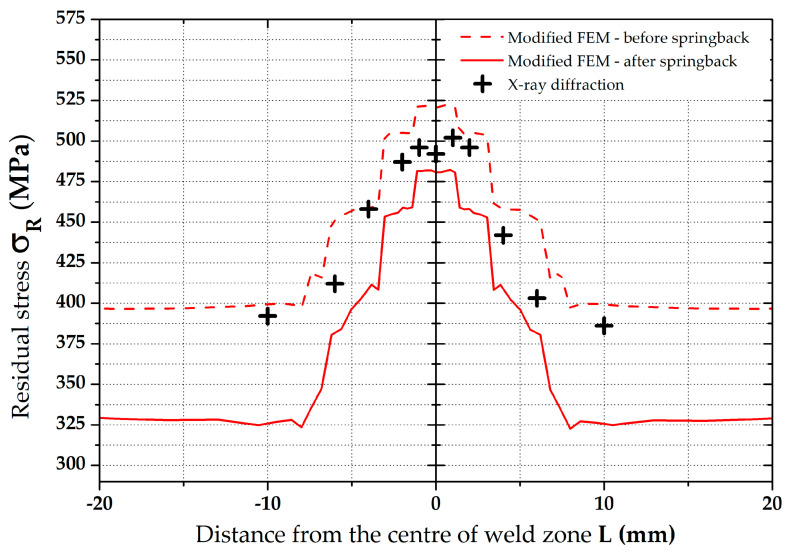
Comparison of residual stresses (von Mises)—XRD vs. modified FEM.

A basic evaluation of this modified FEM was performed as in the previous case. Thus, data in the position 0° were again graphically illustrated—see Figure 21. In the other zones, only the average values were subsequently compared, as shown in Figure 23.

## 4. Discussion

The development of new hardware and software IT in recent years has enabled acceleration in all sectors of production processes’ numerical support. There are still more and more complex mathematical models being introduced, which take into account a considerable number of parameters in the numerical computations. The accuracy of these computations for the individual parts of the technological processes is already at a high level and the obtained results allow us to design complex production processes and also to predict potential problems. The actual trend in the field of industrial IT lies in the creation of a so-called virtual factory, where the chain of all production technologies is numerically simulated. Due to the often completely different requirements for key input parameters of the individual technological processes (e.g., the physical principle of the process, requirements for the finite element mesh, complexity and type of mathematical model, etc.), the challenge of this concept lies primarily in the data compatibility in light of the input and output values required for the individual technological operations. The identification and possible elimination of problems fundamentally affecting the chain of subsequent technological processes are also very needed. Despite the problems mentioned above, the first studies have shown that the concept of a virtual factory, in combination with appropriately adjusted sensors and monitoring of real processes, ensures significant time and cost savings compared to conventional methods, and further development of this concept is expected.

When designing the shape and dimensions of machine components, designers often rely on tabulated values of mechanical properties. However, knowledge of the distribution and magnitude of internal residual stresses, which are, to a large extent, influenced by the history of component production, is also important for the correct functioning and lifetime of the given components. Such manufactured components must therefore be taken as a chain of successive technological procedures. In this paper, the possibility to numerically predict the magnitude of residual stresses in a formed, seamed pipe is presented, taking into account both the welding and forming processes. Both technological processes introduce internal material instability in the form of residual stresses, and, in particular, the welding technology introduces high values of residual stresses that are significantly localized in the weld zone. The uniqueness and novelty of the presented solution for the numerical prediction of residual stresses in a formed, seamed pipe lie in the use of a so-called virtual chain of two consecutive technological operations: welding and forming, realized using specialized software for welding (Sysweld) and sheet metal forming (PAM-Stamp 2G). In contrast to the commonly presented research studies dealing with the numerical simulation of welding or forming processes, the submitted paper presents a comprehensive view of the two successive technological processes, and it also respects the related transfer of results between these two software programs.

To compare the residual stress values determined by the numerical simulations with the real ones, it was also necessary to perform experimental measurements of residual stresses. For this purpose, the X-ray diffraction method was chosen. Four locations at the transition zone between the deformed and undeformed parts of the pipe were chosen for this analysis. The weld zone was taken as the starting position (0°) and three other positions were located at 90° rotations to this reference point. Due to the technical parameters of the X-ray diffraction equipment, it was not possible to measure areas with a diameter lower than 1 mm. Thus, the residual stress analysis was always performed at distances of 1, 2, 4, 6 and 10 mm from the center of the given position. Results from such residual stress analysis revealed a high-stress gradient in the weld zone and almost equal residual stress values in the parent metal in positions 90° and 270°. Average residual stress values measured at these locations (376.9 MPa and 376.0 MPa, respectively) can be considered practically identical from a technical point of view. The position 180° (opposite to the weld) shows that residual stress values are lower by approx. 30 MPa, which is about a 7% decrease compared to positions 90° and 270°. This difference can be explained by the fact that in the zone opposite to the weld is a processed sheet that is fixed during the roll bending process, and thus the residual stresses are partially suppressed. The experimentally determined values of the residual stress values were then compared with the values obtained from the numerical simulations.

To prove the possible positive effect of using a combination of two software to determine residual stresses, two calculation strategies were chosen. In the first case, the standard approach was used in practice, where the numerical simulation of the seamed pipe forming process was performed using only the PAM-Stamp 2G software. In this case, the seamed welded pipe was taken as an inhomogeneous material with different mechanical properties around the weld zone, regardless of any internal stresses introduced into the pipe by the welding process. In this study, this strategy is termed “Standard FEM”. Compared to the common practice, where seamed pipe forming problems are calculated with a homogeneous deformation model neglecting the effect of welding, this approach certainly gives more accurate results. Thus, in order to achieve a higher degree of accuracy in the results computed by the numerical simulation of the forming process, the seamed pipe was divided into several zones (PM, HAZ, HAZ + W and W), where different mechanical properties were taken into account. Thus, to properly define the material cards for the numerical simulations of the forming process in the software PAM-Stamp 2G, it was necessary to measure the mechanical properties of these individual zones. Although the widths of the samples for the static tensile test corresponding to the individual weld zones were chosen to be only 2 mm (see Figure 2), it is clear that it is not possible to strictly separate the individual weld zones when measuring the mechanical properties. The resulting mechanical values in the individual zones thus represent the average properties of the given volume. However, by the nature of the performed experiment and numerical simulation, it is never possible to measure or numerically compute a continuous change in the mechanical properties around the weld.

The determined change in mechanical properties measured by static tensile testing in the weld zone is evident from Table 1 and Figure 3, which graphically illustrates that there was a 44% increase in yield strength, a 22% increase in ultimate tensile strength and that the ductility was lowered by about 6 times compared to the parent metal. As already mentioned above, in the case of the first strategy of residual stress numerical calculation, only a numerical simulation of the forming process was performed without considering the previous welding process. For the finite element computation, a constant size of all elements was chosen, namely 0.6 × 1 × 1 mm (circumferential direction × thickness direction × length of pipe), as is shown in Figure 7 and Figure 13. The measured mechanical properties were assigned to the relevant zones of the seamed pipe, also defined were the technological conditions of the forming process and finally, the numerical computation of forming a well by springback was performed. Based on the results of the numerical simulation “Standard FEM”, the magnitudes of the residual stresses after springback were determined in the relevant zones.

In the case of the second strategy, the numerical prediction of residual stresses was implemented as a chain of two consecutive numerical simulations: the welding and forming processes. In this second strategy, termed in this study as “Modified FEM”, the transfer of residual stresses results between Sysweld (influence of the welding process) and PAM-Stamp 2G (influence of the forming process) is thus taken into account. At first, a numerical simulation of the welding process was performed, and as a partial result, there was a determination of the stress distribution around the weld. These values were subsequently transferred to the PAM-Stamp 2G. Thus, before the numerical simulation of the forming process, the seamed pipe had already been “loaded” by the residual stresses arising from the welding process. This resulted in the data transfer in the following chain: the numerical simulation of the welding in the Sysweld environment, with the subsequent numerical simulation in the PAM-Stamp 2G environment. The basic condition for the possible data transfer between the software mentioned above was the use of the same finite element mesh for the analyzed pipe. Considering the specific requirements for creating the finite element mesh for the numerical simulation of the welding in Sysweld presented in Chapter 3 (Figure 7, in particular, the high density of the mesh in the weld zone), the same finite element mesh as the one used in Sysweld was chosen for the forming process. Compared to the first numerical simulation strategy (Standard FEM), where only the numerical simulation of the forming process with a constant size of mesh elements (0.6 × 1 × 1 mm) was performed, in the second strategy (Modified FEM), a deformation mesh from Sysweld with variable element size was used for the forming process. Such deformation is significantly different compared to the originally used deformation mesh (“uniform” one). When comparing the two applied strategies of the deformation mesh (Standard and Modified FEM) for numerical simulations of the forming process, it can be seen that in the weld zone, the size of the deformation mesh elements was significantly reduced from the original 1 mm up to 0.3 mm in the circumferential direction from the weld, and in the thickness direction it was from 0.6 mm up to 0.3 mm in the Modified FEM strategy. In the length direction, the element size was again 1 mm. In the area of the base material, mesh elements with a size of 3 × 1 × 1 mm were chosen in the case of the Modified FEM to lower the computational time. Elements of this size were located at a sufficient distance from the welding line (approx. 11 mm) and did not significantly affect the calculation of the welding process. When the results of the numerical simulation were subsequently transferred from Sysweld to the numerical simulation of the forming process in PAM-Stamp 2G, the selected maximum element size of 3 × 1 × 1 mm ensured a sufficiently stable and accurate calculation of the forming process. In light of the relative compatibility of the two software used, the effort to achieve the most accurate results and at the same time an acceptable computational time, it would be possible to consider refining the mesh in the zone of the parent metal from a value of 3 × 1 × 1 mm to a value of 1.5 × 1 × 1 mm. The refinement of the mesh elements in the weld zone no longer makes sense in terms of results accuracy.

**Figure 22:**
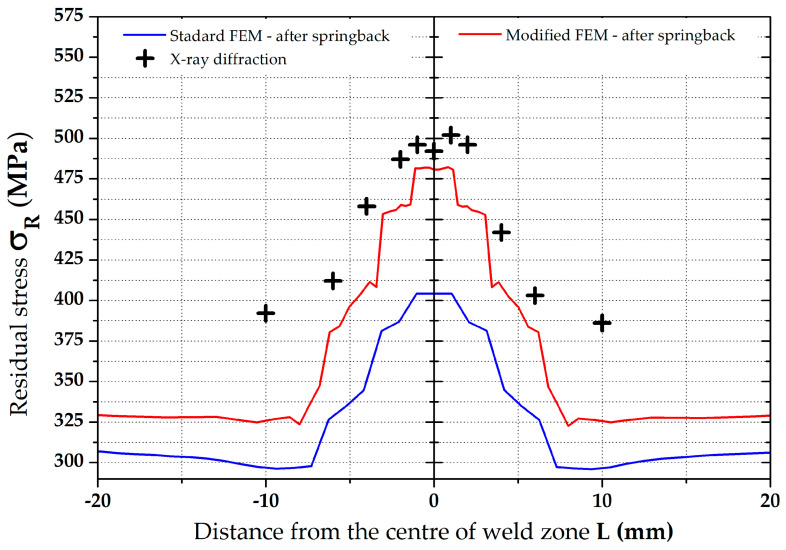
Comparison of residual stresses (von Mises)—XRD vs. standard and modified FEM.

Results of the residual stress distributions in the weld zone from both numerical simulation strategies (both Standard and Modified FEM) can be clearly seen in Figure 22, where measured residual stress values from the XRD method are also shown. To compare these values of the residual stresses determined both by the numerical simulation and experimentally, only the residual stress values after springback were considered because they correspond to the actual condition during the pipe production. From the results presented in the comparison in Figure 22, it is evident that a significantly higher agreement with the numerical simulation with experiment was achieved for the FEM computation with respect to the residual stresses after welding as input data (modified FEM). For better visibility, Table 7, showing percentage differences (for both FEM strategies), was created for the weld zone. Experimentally determined residual stress values were taken as the basis (100%) for the calculation of these differences. From Table 7, it can be seen that, for the standard FEM strategy, the average difference is 20.6%. For the second FEM strategy (modified FEM), the average difference is 7.4%. From the results, it is also evident that the highest differences for both FEM strategies are in the zone of parent metal. On the contrary, the lowest differences were determined in the weld zone.

In the positions 90°, 180° and 270°, only the average values of the residual stresses were compared with each other and are graphically shown in Figure 23. An overview of these results is, together with percentage differences, given in Table 8.

The results given in Table 8 show that the influence of the computational model is significantly lower in the presented zones than in the weld zone. Both strategies of numerical computation (standard and modified FEM) revealed lower values than the experimentally measured residual stresses. This fact is probably given by the reality that the residual stress values measured via the XRD were already influenced by the roll bending technology as a part of the production chain, where tensile stresses are generated on the pipe surface. The numerical simulation of the welding process can provide information about the stresses around the weld zone but does not substantially affect the remaining parent metal of the tested pipe in terms of the internal stress state. For this reason, in the Sysweld software, zones of the parent metal reveal only minimal residual stresses in the order of ones of MPa, which were subsequently transferred to PAM-Stamp 2G. If the distribution of residual stresses was to be virtually refined, the chain would have to take into account an initial roll bending process, then a welding process and finally the metal forming of the tested pipe.

## 5. Conclusions

The major aim of the research presented in this paper was to implement the concept of the virtual factory for numerical simulations of two successive production technologies: welding (software: Sysweld) and metal forming (software: PAM-Stamp 2G). In accordance with the main expected outcome, there was an increase in the accuracy of these FEMs. The actual experimental part consisted of welding the seamed pipe and the subsequent formation of its end part. The determined magnitudes of the residual stresses were used as comparison criteria, and their actual values were measured using the XRD. Subsequently, two different FEM strategies were performed, which differed in terms of considering the welding process. In the first case (standard FEM), there was no implementation of the residual stresses after the welding in the computation of the forming process. Only the different values of mechanical properties in the investigated zones (W, W + HAZ, HAZ and PM) were taken into account. The influence of the welding-induced residual stresses was only taken into account in the case of the so-called modified FEM strategy. However, a rather significant modification within the numerical simulation of the forming process was needed in this case. Subsequently, the results of the two strategies were compared, not only with each other but also with the real residual stress values. The following conclusions can be taken from the measured and computed results:

- It was not possible to continuously measure the distribution of residual stresses in local areas of the weld zone and HAZ using the XRD method. Due to the technical parameters of the used equipment, the minimum distance between the measured points was 1 mm;
- In light of the seamed pipe numerical simulation accuracy, it is suitable to divide the pipe into several zones, with respect to the expected change in mechanical properties in the weld zone;
- No good agreement was achieved between the values of residual stresses from FEM and the experimentally measured ones by using the standard FEM definition without taking into account the previous history of the pipe production (i.e., welding in this case). Around the whole circumference of the pipe, the FEM residual stress values were approximately 20% lower than the experimentally measured values;
- More accurate agreement with the experimental results was achieved using a modified FEM method with respect to the residual stresses from the previous welding technology. In the weld zone and HAZ, the difference was only about 5%, and in the parent metal, the difference was about 16%;
- The submitted solution revealed both the possibility and suitability of using such a complex FEM approach in the production process, in this case, welding and forming;
- Both of the used FEM approaches do not provide sufficiently accurate results for the residual stress distribution in the parent material zone. This is probably because it was not taken into account the complete history of the pipe production, so the effect of roll bending was not considered;
- Despite the fact that, for numerical simulations, software programs from the same manufacturer were used, it was very difficult to ensure data compatibility between them (chain Sysweld and PAM-Stamp 2G). Considering the effect of roll bending would require the use of the following chain: PAM-Stamp 2G–Sysweld–PAM-Stamp 2G, which is currently practically impossible to achieve.

## Figures and Tables

**Table 1:** Basic material properties of the tested material S355JR.

Area	*R*_*p*0.2_ (MPa)	*R_m_* (MPa)	*A_g_* (%)	*A*_40mm_ (%)	*E* (MPa)
PM	392.5	491.7	12.3	20.8	204,587
HAZ	433.4	513.6	9.8	17.9	202,771
W + HAZ	474.2	522.1	4.6	8.1	203,744
W	564.7	600.2	2.2	3.2	201,196

**Table 2:** Stress–strain curve approximation constants from the static tensile test.

Zone	K (MPa)	n (-)	ε_0_ (-)
PM	707.4	0.11701	0.00740
HAZ	673.1	0.07551	0.00346
W + HAZ	658.2	0.05886	0.00282
W	697.7	0.03264	0.00121

**Table 3:** X-ray diffraction—basic parameters.

Parameter	Value
Exposure time	1 s
Collimator	Ø 1 mm
X-ray oscillation	±5°
Number of angle β inclinations	11
Number of exposure repetitions for each measured diffraction maximum	15

**Table 4:** Measured values of the residual stresses σ_R_ (MPa) in the relevant points.

Distance from Center of Weld	Measured Position (°)			
	0° (Weld Zone)	90°	180°	270°
−10 mm	392	378	356	388
−6 mm	412	371	347	371
−4 mm	458	383	341	382
−2 mm	487	369	352	376
−1 mm	506	382	361	374
0	492	376	355	367
1 mm	502	370	344	369
2 mm	496	384	349	382
4 mm	442	375	339	372
6 mm	403	381	342	379
10 mm	386	376	338	370

**Table 5:** Standard FEM: comparison of the residual stress (von Mises) values σ_R_ (MPa) and XRD.

Distance from the Center of Weld Zone	Measured Position (°)							
	0°		90°		180°		270°	
	XRD	FEM	XRD	FEM	XRD	FEM	XRD	FEM
−10 mm	392	297	378	312	356	316	388	312
−6 mm	412	326	371	311	347	316	371	311
−4 mm	458	344	383	311	341	316	382	312
−2 mm	487	386	369	312	352	317	376	311
−1 mm	506	404	382	312	361	317	374	311
0	492	404	376	312	355	316	367	312
1 mm	502	404	370	312	344	317	369	311
2 mm	496	386	384	312	349	317	382	311
4 mm	442	344	375	311	339	317	372	312
6 mm	403	326	381	311	342	317	379	311
10 mm	386	297	376	312	338	316	370	312

**Table 6:** Modified FEM—comparison of the residual stress (von Mises) values σ_R_ (MPa) and XRD.

Distance from the Center of Weld Zone	Measured Position (°)							
	0°		90°		180°		270°	
	XRD	FEM	XRD	FEM	XRD	FEM	XRD	FEM
−10 mm	392	324	378	334	356	336	388	333
−6 mm	412	380	371	333	347	336	371	334
−4 mm	458	403	383	334	341	335	382	334
−2 mm	487	459	369	335	352	335	376	333
−1 mm	506	481	382	334	361	336	374	333
0	492	481	376	335	355	337	367	334
1 mm	502	481	370	334	344	336	369	333
2 mm	496	458	384	335	349	336	382	333
4 mm	442	402	375	334	339	337	372	334
6 mm	403	380	381	333	342	337	379	334
10 mm	386	325	376	334	338	336	370	333

**Table 7:** Comparison of all determined residual stresses (von Mises)—position 0.

Distance from the Center of Weld Zone	XRD	Standard FEM	Difference	Modified FEM	Difference
	(MPa)	(MPa)	(%)	(MPa)	(%)
−10 mm	392	297	24.2	324	17.3
−6 mm	412	326	20.9	380	7.8
−4 mm	458	344	24.9	403	12.0
−2 mm	487	386	20.7	459	5.7
−1 mm	506	404	20.2	481	4.9
0	492	404	17.9	481	2.2
1 mm	502	404	19.5	481	4.2
2 mm	496	386	22.2	458	7.7
4 mm	442	344	22.2	402	9.0
6 mm	403	326	19.1	380	5.7
10 mm	386	297	23.1	325	15.8

**Table 8:** Comparison of all determined residual stresses (von Mises)—positions 90°, 180° and 270°.

Measured Position	XRD	Standard FEM	Difference	Modified FEM	Difference
	(MPa)	(MPa)	(%)	(MPa)	(%)
90°	377.0	311.7	17.3	334.2	11.3
180°	344.5	316.7	8.1	336.5	2.3
270°	373.2	311.5	16.5	333.5	10.6

## Data Availability

Not applicable.
